# Genome sequences of seven *Streptomyces* isolates for genome prospecting

**DOI:** 10.1128/mra.00117-26

**Published:** 2026-05-29

**Authors:** Donovan Babka, Ava Eagan, Bianca Gonzalez, Avary Torres, Amara Ueligitone, Diego Valderrama, Iminii Wilmer, Imen Nouioui, Markus Göker, Alex Spundec, Stephan Ritter, Danielle Graham, Natalia Ivanova, Rekha Seshadri, Craig Stephens

**Affiliations:** 1 Department of Biology, Santa Clara University 7162, Santa Clara, California, USA; 2 Leibniz Institute DSMZ, German Collection of Microorganisms and Cell Cultures 28351, Braunschweig, Germany; 3 Lawrence Berkeley National Laboratory, DOE Joint Genome Institute 118576, Berkeley, California, USA; University of Strathclyde, Glasgow, United Kingdom

**Keywords:** *Streptomyces*, genomics, antibiotic synthesis

## Abstract

We report genome sequences for seven *Streptomyces* isolates from the Leibniz Institute DSMZ culture collection, each with more than 20 predicted biosynthetic gene clusters that may be resources for discovery of novel antibiotics and other bioactive products.

## ANNOUNCEMENT

*Streptomyces* species (*Bacteria*, *Actinomycetota*, *Actinomycetes*, *Kitasatosporales*, *Streptomycetaceae*) are soil-dwelling gram-positive filamentous bacteria recognized for synthesizing diverse secondary metabolites (1, 2). The Leibniz Institute DSMZ culture collection has collaborated with the United States (US) Department of Energy’s Joint Genome Institute (JGI) on the “*Sequencing the genomes of 1000 actinobacteria strains*” project for large-scale development of genomic resources that could support the discovery and production of new antibiotics and other bioactive compounds (2). The seven genome sequences presented here are isolates from the DSMZ collection for which long-read sequencing had generated near-complete assemblies.

*Streptomyces* cultures were revived from freeze-dried cells and grown at DSMZ in liquid media based on the strain preference (Table 1) (3) and incubated for 7 days at 28°C with shaking at 120 rpm. Genomic DNA was extracted at DSMZ using the MasterPure Gram-Positive DNA Purification Kit (Biosearch Technologies). An input of 1 μg of genomic DNA was sheared to approximately 10 kb using the Megaruptor 3 (Diagenode). The sheared DNA was treated with exonuclease to remove single-stranded ends, DNA damage repair enzyme mix, end-repair/A-tailing mix and ligated with barcoded overhang adapters using SMRTbell Express Template Prep Kit 2.0 (Pacific Biosciences). Up to 16 libraries were pooled in equimolar concentrations and purified with AMPure PB Beads (PacBio). PacBio Sequencing primer was then annealed to the SMRTbell template library, and sequencing polymerase was bound to them using Sequel II Binding Kit 2.0. The prepared SMRTbell template libraries were then sequenced on a PacBio Sequel II sequencer (4) using 8M v1 SMRT cells and Version 2.0 sequencing chemistry with 1 × 900 sequencing movie run times. Read quality control, error correction, and adapter trimming were performed by SMRT Link (v 8.0.0.78867) software under default conditions. Reads were additionally filtered for collapsed smartbells (missing adapters) using icecreamfinder.sh, a script in the BB Tools suite (v38.87) (5). Default parameters were used for all software unless otherwise specified. Filtered subreads and Circular Consensus Sequence (CCS) reads were used as input for *de novo* assembly using either Flye (v2.8.1) (6), with the parameters “–asm-coverage 100 –plasmid –genome-size 5,000,000 -t 64 –pacbio-raw,” or Pacific Biosciences HGAP4 software (v. smrtlink/8.0.0.80529) (7). Coverage and other statistics for final assemblies, which consisted of one to five linear contigs, are shown in Table 1. Sequences were annotated by the IMG annotation pipeline (version 5.0.23) (8). CheckM2 (v1.0.2) confirmed greater than 99.6% completeness for all assemblies (9). Most of these draft assemblies have one large contig representing all or the vast majority of the assembly, but it has not yet been independently verified that these represent complete chromosome replicons. Likely plasmid contigs were identified by GeNomad (v. 1.1) in four of the genomes, as indicated in Table 1 (10).

**TABLE 1:** Genomic analysis of *Streptomyces* isolates presented here

Sequenced isolate	DSM 40229	DSM 40803	DSM 40843	DSM 40167	DSM 41037	DSM 41269	DSM 41600
Species designation^*a*^	*S. griseoviridis*	*S. virginiae* (*S. cinnamonensis*^*b*^)	*S. clavifer*	*Streptomyces* sp.	*S. diastaticus*	*Streptomyces* sp.	*S. demainii*
Source^*c*^ (sample, location)	Soil (Texas, USA)	Soil (Kanegasaki, Japan)	Limed plot and scab on *Solanum tuberosum* (location unknown)	Soil (former Soviet Union)	Unknown	Unknown	Unknown
Growth conditions^*d*^	ISP 2 (DSMZ 987), 28°C, 7 days	ISP 2, 28°C, 7 days	DSMZ 252, 28°C, 7 days	ISP 2, 28°C, 7 days	ISP 2, 28°C, 7 days	ISP 2, 28°C, 7 days	ISP 2, 28°C, 7 days
Total raw sequence reads (ave read length)	1,240,838 (4,796 bp)	1,171,095 (4,917 bp)	618,908 (5,232 bp)	765,326 (5,662 bp)	1,952,711 (4,749 bp)	1,505,523 (5,167 bp)	1,030,103 (6,246 bp)
# of filtered reads assembled (ave read length)	206,560 (4,830 bp)	202,391 (4,950 bp)	190,031 (5,255 bp)	175,558 (5,707 bp)	208,604 (4,799 bp)	191,230 (5,225 bp)	159,356 (6,272 bp)
Assembly software	Flye (v 2.8.1)	Flye (v 2.8.1)	HGAP4 v. smrtlink/8.0.0.80529	HGAP4 v. smrtlink/8.0.0.80529	Flye (v 2.8.1)	HGAP4 v. smrtlink/8.0.0.80529	Flye (v 2.8.1)
Genome coverage	120.9×	113×	123×	116.8×	143×	118.4×	91×
Contigs (size)	One contig (7,872,322 bp)	One contig (8,588,732 bp)	Two contigs (6,644,253 bp; 1,058,841 bp^*e*^)	Two contigs (7,997,812 bp; 180,019 bp^*e*^)	Two contigs (6,660,302 bp; 40,395 bp)	Four contigs (7,875,433 bp; 123,356 bp^*e*^; 92,112 bp^*e*^; 11,123 bp^*e*^)	Five contigs (6,271,230 bp; 4,120,260 bp; 50,010 bp; 18,527 bp^*e*^; 82,325 bp)
Contig N50	7,872,322 bp	8,588,732 bp	6,644,253 bp	7,997,812 bp	6,660,302 bp	7,875,433 bp	6,271,230 bp
GC (%)	73.2	72.3	71.3	71.9	73.5	72.5	71.9
Total CDS	6,855	7,777	6,705	7,427	5,632	7,118	8,775
JGI accession #	2923575836	2918290305	2918298223	2926612960	2926786735	2926068284	2923609139
NCBI accession #	JAURUD000000000.1	JAGINR000000001.1	JAGINS010000000	JAUSUS000000000	JAUSVC000000000	JAUSRK000000000	JAURUE000000000
SRA accession #	SRX20649993	SRX14800871	SRX14800870	SRX20650027	SRX20650028	SRX20650031	SRX14800875
Predicted biosynthetic gene clusters (SMC ID)^*f*^	27 (1163691)	34 (1184840)	31 (146046)	33 (1163863)	28 (1163876)	35 (1163764)	49 (1163694)

^*a*^ Species designation in Leibniz Institute—DSMZ—German Collection of Microorganisms and Cell Culture GmbH record.

^*b*^ Original strain designation of *S. cinnamonensis* was later reclassified by Komaki and Tamura (11) to be a heterotypic synonym of *S. virginiae*. Some accession records still refer to the strain as *S. cinnamonensis*.

^*c*^ Sources according to the Leibniz Institute—DSMZ BacDive database.

^*d*^ Media recipes can be found in MediaDive (3).

^*e*^ Predicted plasmids according to JGI-IMG annotation pipeline employing GeNomad (10).

^*f*^ Links are provided to detailed AntiSMASH results through the JGI Secondary Metabolism Collaboratory website (SMC ID shown) (12).

AntiSMASH (v.7.1.0) predictions of biosynthetic gene clusters (BGCs) are shown in Table 1 (13). These included polyketide synthases, non-ribosomal peptide synthetases, and ribosomally synthesized post-translationally-modified peptide (RiPP) gene clusters often associated with antibiotic production. The large number and diversity of BGCs in these genomes may offer abundant opportunities for discovery of novel bioactive structures and functions.

## Data Availability

The raw sequencing data and annotated genome sequences described here are freely available for analysis at the links shown in Table 1. Note that the IMG and NCBI annotations can be slightly different, as they use different annotation pipelines.
